# Molecular and phenotypic spectrum of cardio-facio-cutaneous syndrome in Chinese patients

**DOI:** 10.1186/s13023-023-02878-0

**Published:** 2023-09-11

**Authors:** Biyun Feng, Xin Li, Qianwen Zhang, Yirou Wang, Shili Gu, Ru-en Yao, Zhiying Li, Shiyang Gao, Guoying Chang, Qun Li, Niu Li, Lijun Fu, Jian Wang, Xiumin Wang

**Affiliations:** 1 Department of Endocrinology, Metabolism and Genetics, Shanghai Children’s Medical Center, Shanghai Jiao Tong University School of Medicine, Shanghai, 200127 China; 2 Department of Genetic Molecular Diagnostic Laboratory, Shanghai Children’s Medical Center, Shanghai Jiao Tong University School of Medicine, Shanghai, 200127 China; 3 Department of Cardiology, Shanghai Children’s Medical Center, Shanghai Jiao Tong University School of Medicine, Shanghai, 200127 China

**Keywords:** Cardio-facio-cutaneous syndrome, RASopathy, *BRAF*, *MAP2K1/2*

## Abstract

**Background:**

Cardio-facio-cutaneous (CFC) syndrome is a RASopathy subtype that presents with unique craniofacial dysmorphology, congenital heart disease, dermatologic abnormalities, growth retardation, and intellectual disability. This study describes the phenotypic spectrum of CFC in China and its association with CFC syndrome gene variants.

**Results:**

Twenty Chinese CFC patients, aged 0.6–9.5 years old, were included in this study and their clinical phenotypic spectrum was compared with that of 186 patients with CFC from non-Chinese ethnicities. All 20 Chinese patients with CFC carried de novo heterozygous *BRAF*, *MAP2K1*, and *MAP2K2* variants. Two novel variants were detected and consistently predicted to be deleterious using bioinformatic tools. The clinical features of CFC in the Chinese patients included hypertrophic cardiomyopathy (2/20, 10%), pulmonary valve stenosis (2/20, 10%), curly or sparse hair (7/20, 35%), epilepsy (1/20, 5%), and hypotonia (10/20, 50%); these features were less frequently observed in Chinese patients than non-Chinese patients (*p* < 0.05). In contrast, feeding difficulties (19/20, 95%) were more frequently observed in the Chinese patients. Absent eyebrows and severe short stature were more common in patients with *BRAF* variants than in those with *MAP2K1/2* variants. Facial recognition software was used to recognize most CFC patients using artificial intelligence.

**Conclusion:**

This study identified novel and common variants in our cohort of 20 Chinese patients with CFC. We uncovered differences in clinical features between Chinese and non-Chinese patients and detected genotype–phenotype correlations among the *BRAF* and *MAP2K1/2* variant subgroups. This is the largest cohort of Chinese CFC patients to our knowledge, providing new insights into a subtype of RASopathy.

**Supplementary Information:**

The online version contains supplementary material available at 10.1186/s13023-023-02878-0.

## Background

Cardio-facio-cutaneous syndrome (CFC; MIM#115150) was first reported by Reynolds et al. in 1986 [1]. CFC is an autosomal dominant disorder characterized by dysmorphic craniofacial features, congenital heart disease, dermatologic abnormalities, failure to thrive, gastrointestinal dysfunction, neurocognitive delay, and epilepsy [1]. The prevalence of CFC in Japan is estimated at 1 in 810,000 individuals. At present, there are few case reports of CFC patients in China; large-scale cohort studies of Chinese patients are still lacking, making it difficult to summarize the common features of CFC in China.

CFC is a RASopathy whose phenotype overlaps with Noonan syndrome (NS, MIM#163950) and Costello syndrome (CS, MIM#218040), which are caused by germline variants in components of the RAS-mitogen-activated protein kinase (MAPK) signaling pathway. Patients with CFC harbor activating missense variants in *BRAF* (MIM#115150)*, MAP2K1* (MIM#615279)*, MAP2K2* (MIM#615280), and *KRAS* (MIM#615278), most of which are de novo variants [2]. Heterozygous *BRAF* variants (~ 75%) constitute the majority of CFC cases, whereas variants in the *MAP2K1* or *MAP2K2* genes (~ 25%) and *KRAS* (< 2%) also cause CFC. The *BRAF* gene encodes the BRAF protein (serine/threonine protein kinase), whose germline variants are located in the cysteine-rich and protein kinase domains. *BRAF* variants that cause CFC syndrome are found in exons 6, 11, 12, 13, 14, 15, and 16. *MAP2K1/2* encodes mitogen-activated protein kinase 1/2 (MAP2K1/2), which activates extracellular signal-regulated kinases 1/2 (ERK1/2). Variants in *MAP2K1/2* occur in exons 2, 3, 6 or 7 [3]. Recently, variants of *YWHAZ* have been reported to exhibit CFC-like manifestations [4]. Nevertheless, in some patients with CFC, no causative gene variants have been identified [5].

The diagnosis of CFC is based on clinical suspicion and genetic testing, including next-generation sequencing (NGS) [1]. The CFC index, as a clinical and objective method, was first proposed in 2002 to identify and differentiate CFC from other phenotypically similar genetic syndromes; however, it is not widely used [6]. In 2014, an international group of experts developed international consensus diagnostic criteria for CFC pediatricians and other care providers. Artificial intelligence (AI) tools, such as DeepGestalt (Face2Gene, FDNA Inc., Boston, MA), have improved facial recognition of genetic syndromes and can achieve > 90% top-10 diagnostic accuracy in correctly identifying a genetic syndrome [7].

Here, we report the clinical and genetic spectrum of 20 Chinese patients with CFC, which, to our knowledge, is the largest CFC cohort studied in China. Furthermore, genotype–phenotype correlations were analyzed, and the phenotypes of CFC patients in China were compared to those of patients in Canada. Finally, to explore new diagnostic tools, we used AI tools to improve the diagnostic rate.

## Results

### Clinical data

**Fig. 1:**
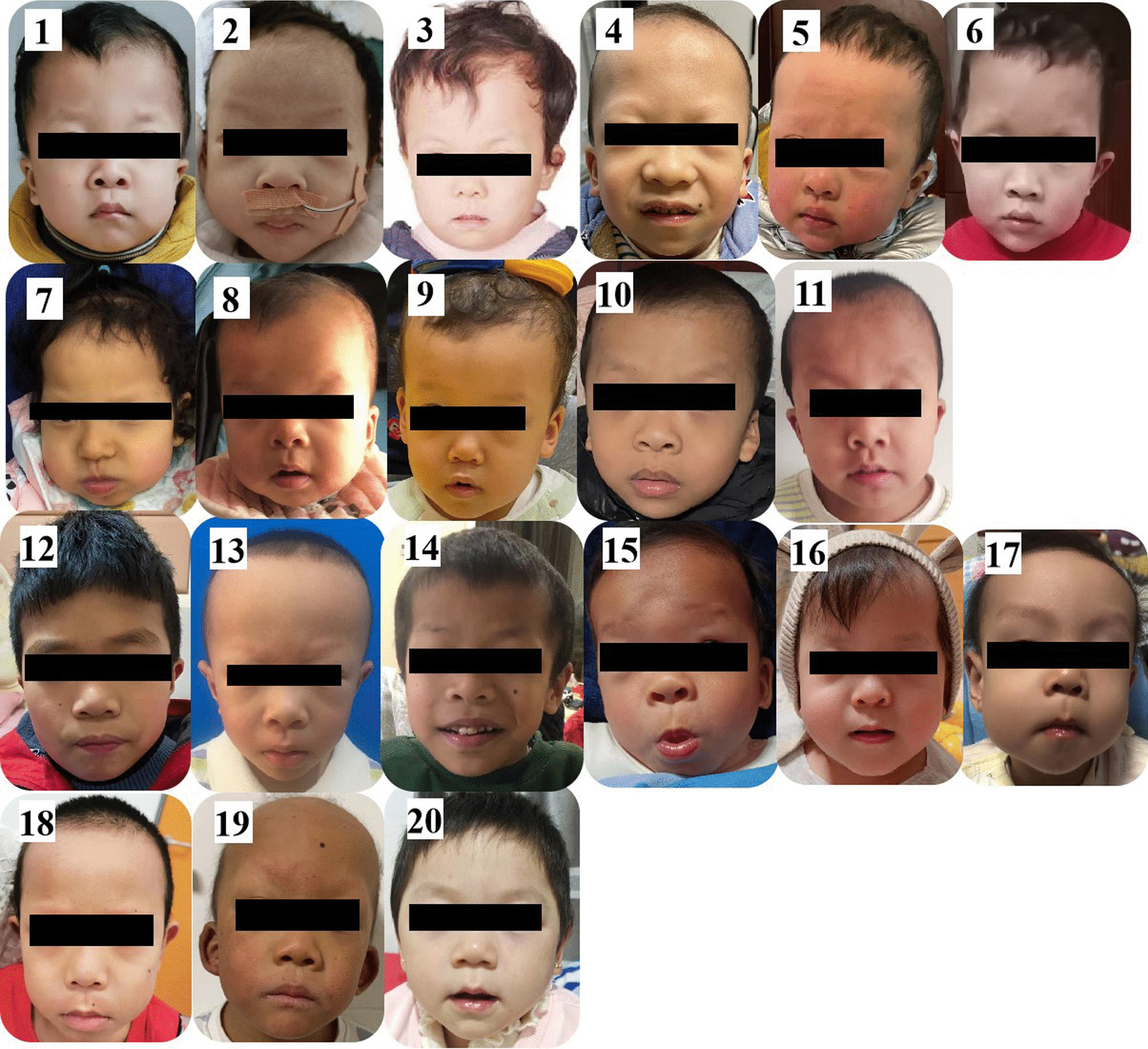
Facial images of CFC patients in this cohort: 1–11 BRAF mutations; 12–18 MAP2K1 mutations; 19–20 MAP2K2 mutations

Twenty patients aged from 0.6 to 9.5 years old (11 male and 9 female) genetically diagnosed with CFC were included in this study. One patient was previously reported in 2019 due to cardiovascular traits, while the other 19 patients have not been reported [8]. Facial pictures are shown in Fig. 1.

Phenotypic information is summarized in Table 1 and covered multisystem abnormalities, including craniofacial, dermatological, cardiovascular, skeletal, neurological/developmental, endocrine, gastrointestinal, tumorous, and prenatal manifestations. Outstanding craniofacial features are wide nasal base/anteverted nares, hypoplasia of the eyebrows, macrocephaly, epicanthus and low-set ears, which were found in more than half of the patients. Two-thirds of the patients have a history of congenital heart disease (13/20, 65%), including atrial septal defect, patent foramen ovale, hypertrophic cardiomyopathy, and pulmonary valve stenosis. The most prominent dermatological abnormalities were melanocytic nevi (60%; 12/20), followed by abnormal nail growth, hemangioma, keratosis pilaris, and eczema. Short stature was observed in 70% (14/20) of patients and the average standard deviation (SD) for height was − 3.0 SD, using standard Chinese growth curves. All patients exhibited delayed motor development and intellectual disability. A total of 65% (13/20) of patients suffered from sleep disturbance, with brain MRI abnormalities present in 55% (11/20) of patients, while only one patient had a history of epilepsy. Perinatal findings were polyhydramnios, preterm birth, and increased nuchal translucency. Other clinical findings included pectus excavatum/carinatum, scoliosis, strabismus, hearing impairment, and cryptorchidism. Only one patient had a tumor, which was an inflammatory myeloid neoplasm called Langerhans cell histocytosis. Four patients in our study were treated with growth hormone (GH). One of the patients was GH deficient (patient 4) and two of them (patient 4, patient 6) achieved a satisfactory result in height. None of them suffered side effects following GH treatment.

**Table 1:** Summary of phenotypes of CFC patients with *BRAF, MAP2K1/2* variants

Clinical features	HPO number	N/total (%) of subjects		
		*BRAF* (11)	*MAP2K1/2* (9)	Total (20)
Craniofacial features				
Macrocephaly	HP: 0000256	10	5	15 (75%)
Spare/curly hair	HP:0002212/HP:0008070	5	2	7 (35%)
Epicanthus	HP: 0000286	6	7	13 (65%)
Down-slanting palpebral fissure	HP: 0000494	4	4	8 (40%)
Ptosis	HP: 0000508	1	2	3 (15%)
Hypoplasia of eyebrow	HP: 0100840	11	5	16 (80%)
Ulerythema ophryogenesis	ORPHA: 3406	2	3	5 (25%)
Low-set ears	HP: 0000358	8	5	13 (65%)
Wide nasal base/anteverted nares	HP: 0012810/HP: 0000463	10	7	17 (85%)
Wide mouth	HP: 0000154	1	2	3 (15%)
Congenital heart defects	HP: 0001627	7	6	13 (65%)
ASD	HP: 0001631	3	4	7 (35%)
PFO	HP: 0001655	2	2	4 (20%)
PVS	HP: 0001642	0	2	2 (10%)
HCM	HP: 0001639	2	0	2 (10%)
Short stature	HP: 0004322	9	5	14 (70%)
GH therapy	–	2	2	4 (20%)
Mean SDS (range)	–	− 3.6 (-0.4 ~ -8.3)	− 2.1 (+ 0.5 ~ − 4.8)	− 3.0 (+ 0.5 ~ − 8.3)
Melanocytic nevi	HP: 0000995	8	4	12 (60%)
Keratosis pilaris	HP: 0032152	2	4	6 (30%)
Hemangioma	HP: 0001028	3	4	7 (35%)
Eczema	HP: 0000964	0	3	3 (15%)
Abnormal nail growth	HP: 0030807	5	5	10 (50%)
Pectus excavatum/carinatum	HP: 0000766	7	5	12 (60%)
Scoliosis	HP: 0002650	2	2	4 (20%)
Motor developmental delay	HP: 0031936	11	9	20 (100%)
Intellectual disability	HP: 0001249	7	5	12 (100%)*
Epilepsy	HP: 0001250	0	1	1 (5%)
Hypotonia	HP: 0001252	6	4	10 (50%)
Brain MRI abnormality	HP: 0012747	5	6	11 (55%)
Sleep disturbance	HP: 0002360	8	5	13 (65%)
Strabismus	HP: 0000486	4	4	8 (40%)
Hearing impairment	HP: 0000365	0	3	3 (15%)
Cryptorchidism	HP: 0000028	1	4	5 (25%)
Feeding difficulties	HP: 0011968	11	8	19 (95%)
Tumor	HP: 0002664	0	1	1 (5%)
Increased nuchal translucency	HP: 0010880	1	2	3 (15%)
Pemature birth	HP: 0001622	5	3	8 (40%)
Polyhydramnios	HP: 0001561	8	4	12 (60%)

*ASD* atrial septal defect, *PFO* patent foramen ovale, *PVS* pulmonary valve stenosis, *HCM* hypertrophic cardiomyopathy, *GH* growth hormone

*Means intellectual disability of 12/12 patients can be evaluated

### Comparison with published literature

We used statistical tools to analyze the differences in phenotypes between our 20 Chinese patients and a cohort of 186 non-Chinese patients (Table 2). The results indicated that pulmonary valve stenosis, curly/sparse hair, and epilepsy were less likely to occur in Chinese CFC patients with *BRAF* variants, whereas curly/sparse hair and hypotonia were rare in Chinese patients with *MAP2K1/2* variants. Conversely, feeding difficulties were more prominent in the Chinese patients than the non-Chinese patients. It is worth noting that epilepsy may be underestimated because it can also occur in childhood, late adolescence and even early adulthood [9].

**Table 2:** Comparison of CFC syndrome phenotype between Chinese patients and non-Chinese patients reported in the literature

	*BRAF* n/N (%)			*MAP2K1/2* n/N (%)			Total n/N (%)		
	A	B	*p* value	A	B	*p* value	A	B	*p* value
HCM	2/11 (18)	52/137 (38)	.33^b^	0/9 (0)	11/45 (24)	.18^b^	2/20 (10)	63/182 (35)	.02^a^
PVS	0/11 (0)	64/127 (50)	< .01^a^	2/9 (22)	18/48 (38)	.47^b^	2/20 (10)	82/175 (47)	< .01^a^
ASD	3/11 (27)	36/126 (29)	1.00^b^	4/9 (44)	7/39 (18)	.18^b^	7/20 (35)	43/165 (26)	.43^a^
VSD	0/11 (0)	10/87 (11)	.60^b^	0/9 (0)	4/21 (19)	.29^b^	0/20 (0)	14/108 (13)	.12^b^
Relative a/o macrocephaly	10/11 (91)	63/91 (69)	.17^b^	5/9 (56)	14/21 (67)	.69^b^	15/20 (75)	77/112 (69)	.61^a^
Curly a/o sparse hair	5/11 (45)	121/130 (93)	< .01^b^	2/9 (22)	31/40 (78)	< .01^b^	7/20 (35)	152/170 (89)	< .01^b^
Absent a/o sparse eyebrows	11/11 (100)	92/114 (81)	.21^b^	5/9 (56)	32/41 (78)	.21^b^	16/20 (80)	124/155 (80)	1.00^b^
Keratosis pilaris	2/11 (18)	22/46 (48)	.10^b^	4/9 (44)	4/6 (67)	.61^b^	6/20 (30)	26/52 (50)	.19^a^
Nevi	8/11 (73)	44/105 (42)	.06^b^	4/9 (44)	12/33 (36)	.71^b^	12/20 (60)	56/138 (41)	.15^a^
Hypotonia	6/11 (55)	89/112 (79)	.12^b^	4/9 (44)	32/37 (86)	.02^b^	10/20 (50)	121/149 (81)	< .01^b^
Epilepsy	0/11 (0)	34/111 (31)	.03^b^	1/9 (11)	13/40 (33)	.42^b^	1/20 (5)	47/151 (31)	.02^a^
ID	7/7 (100)	75/75 (100)	NA	5/5 (100)	25/27 (93)	1.00^b^	12/12 (100)	100/102 (98)	1.00^b^
Short stature	9/11 (82)	97/117 (83)	1.00^b^	5/9 (56)	32/41 (78)	.21^b^	14/20 (70)	129/158 (82)	.24^b^
Feeding difficulties	11/11 (100)	35/65 (54)	< .01^b^	8/9 (89)	6/15 (40)	.03^b^	19/20 (95)	41/80 (51)	< .01^a^
Cryptorchidism	1/11 (9)	12/40 (30)	.25^b^	4/9 (44)	8/12 (67)	.40^b^	5/20 (25)	20/52 (38)	.41^a^
Strabismus	4/11 (36)	61/111 (55)	.34^a^	4/9 (44)	22/38 (58)	.49^b^	8/20 (40)	83/149 (56)	.23^a^
Pectus deformity	7/11 (64)	45/102 (44)	.34^a^	5/9 (56)	24/38 (63)	.72^b^	12/20 (60)	69/140 (49)	.48^a^
Scoliosis	2/11 (18)	10/29 (34)	.45^b^	2/9 (22)	3/6 (50)	.33^b^	4/20 (20)	13/35 (37)	.24^a^
Polyhydramnios	8/11 (73)	60/96 (63)	.74^b^	4/9 (44)	25/36 (69)	.25^b^	12/20 (60)	85/132 (64)	.80^a^
Premature birth	5/11 (45)	30/70 (43)	1.00^b^	3/9 (33)	12/23 (52)	.44^b^	8/20 (40)	42/93 (45)	.81^a^

*A* Chinese patients, *B* non-Chinese patients, *ID* intellectual disability, *ASD* atrial septal defect, *VSD* ventricular septal defect, *PVS* pulmonary valve stenosis, *HCM* hypertrophic cardiomyopathy

^a^*p* value, per chi-square test

^b^*p* value, per Fisher's precise test

### Spectrum of variants in Chinese CFC patients

**Fig. 2:**
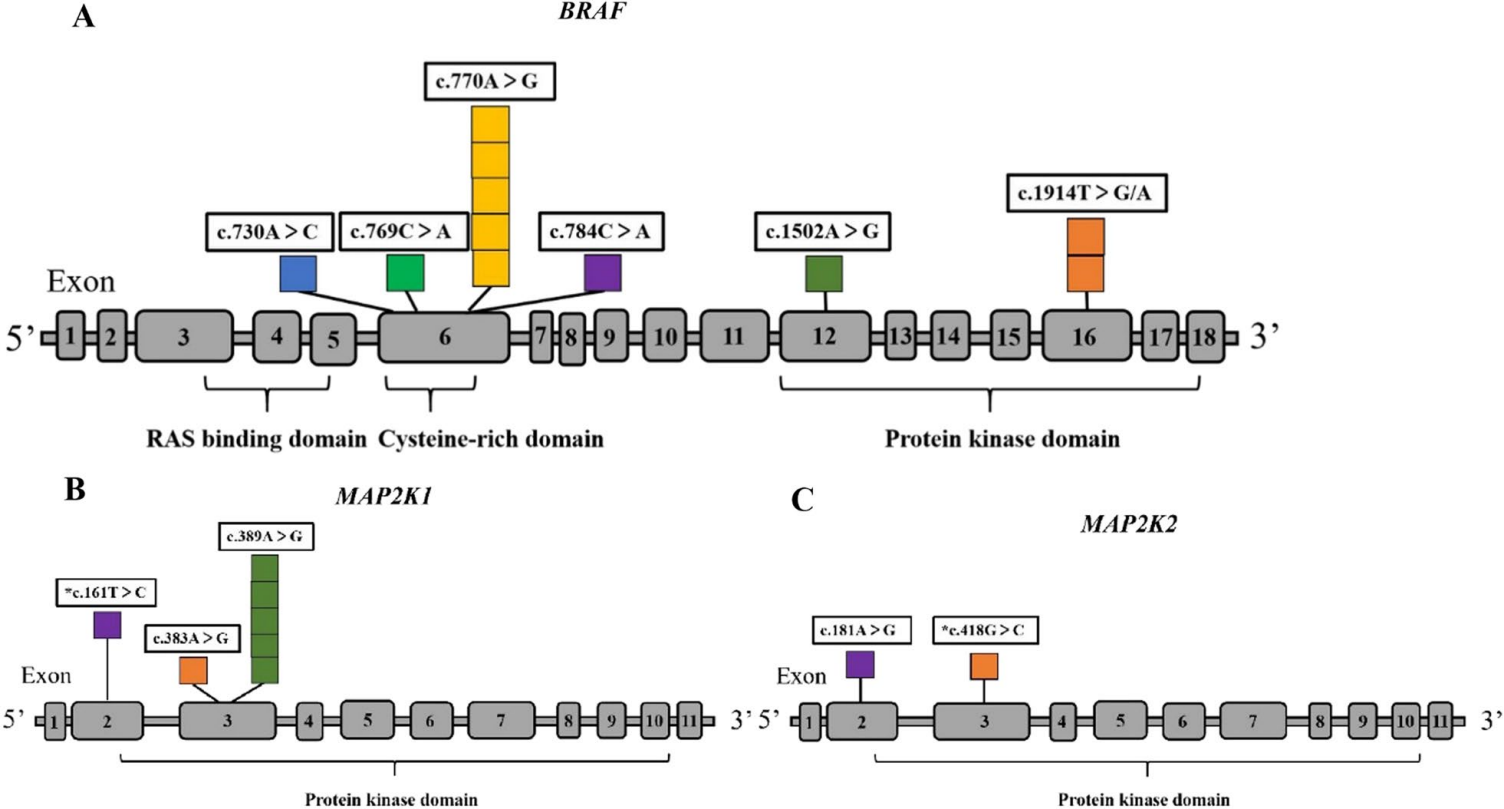
CFC variants identified in this study. Novel variants are shown with *. (**A**) The schematic diagram of the distribution of BRAF mutations in this study. (**B**, **C**) The schematic diagram of the distribution of MAP2K1/2 mutations in this study

A schematic diagram of the variant distribution in this study is shown in Fig. 2. All variants were classified as either likely pathogenic or pathogenic following the American College of Medical Genetics and Genomics (ACMG) guidelines, except the p.Asp140His variant in the *MAP2K2* gene (patient 9), which was classified as having uncertain significance. Two novel variants were identified in this study: one de novo variant (p.Leu54Pro) in *MAP2K1* and one de novo variant (p.Asp140His) in *MAP2K2*. Both variants were located in the functional domain of the protein. All variants, including the two novel variants, were consistently predicted to be deleterious using three bioinformatics tools (Table 3).

**Table 3:** Genotype of the CFC patients and in silico evaluation of novel variants in this study

Gene	Case (n)	Refseq	Exon	DNA change	Protein change	Origin of variant	MAF (gnomAD)	Mutation taster	PolyPhen-2	SIFT	ACMG classification
BRAF	5	NM_004333.5	6	c.770A > G	p.Gln257Arg	de novo	0.000	Disease causing	Probably damaging	Deleterious	Pathogenic
BRAF	2	NM_004333.5	16	c.1914T > G/A	p.Asp638Glu	de novo	0.000	Disease causing	Probably damaging	Deleterious	Pathogenic
BRAF	1	NM_004333.5	6	c.769C > A	p.Gln257Lys	de novo	0.000	Disease causing	Probably damaging	Deleterious	Pathogenic
BRAF	1	NM_004333.5	12	c.1502A > G	p.Glu501Gly	de novo	0.000	Disease causing	Probably damaging	Deleterious	Pathogenic
BRAF	1	NM_004333.5	6	c.784C > A	p.Gln262Lys	de novo	0.000	Disease causing	Probably damaging	Deleterious	Pathogenic
BRAF	1	NM_004333.5	6	c.730A > C	p.Thr244Pro	de novo	0.000	Disease causing	Probably damaging	Deleterious	Pathogenic
MAP2K1	5	NM_002755.3	3	c.389A > G	p.Tyr130Cys	de novo	0.000	Disease causing	Probably damaging	Deleterious	Pathogenic
MAP2K1	1	NM_002755.3	3	c.383G > T	p.Gly128Val	de novo	0.000	Disease causing	Probably damaging	Deleterious	Pathogenic
MAP2K1	1	NM_002755.3	2	*c.161T > C	*p.Leu54Pro	de novo	0.000	Disease causing	Probably damaging	Deleterious	Likely pathogenic
MAP2K2	1	NM_030662.4	2	c.181A > G	p.Lys61Glu	de novo	0.000	Disease causing	Probably damaging	Deleterious	Pathogenic
MAP2K2	1	NM_030662.4	3	*c.418G > C	*p.Asp140His	de novo	0.000	Disease causing	Probably damaging	Deleterious	Uncertain significance

*Means novel variant site; de novo: variants are present in the affected individual but absent from both biological parents' genomes

### Genotype–phenotype correlations in CFC

**Fig. 3:**
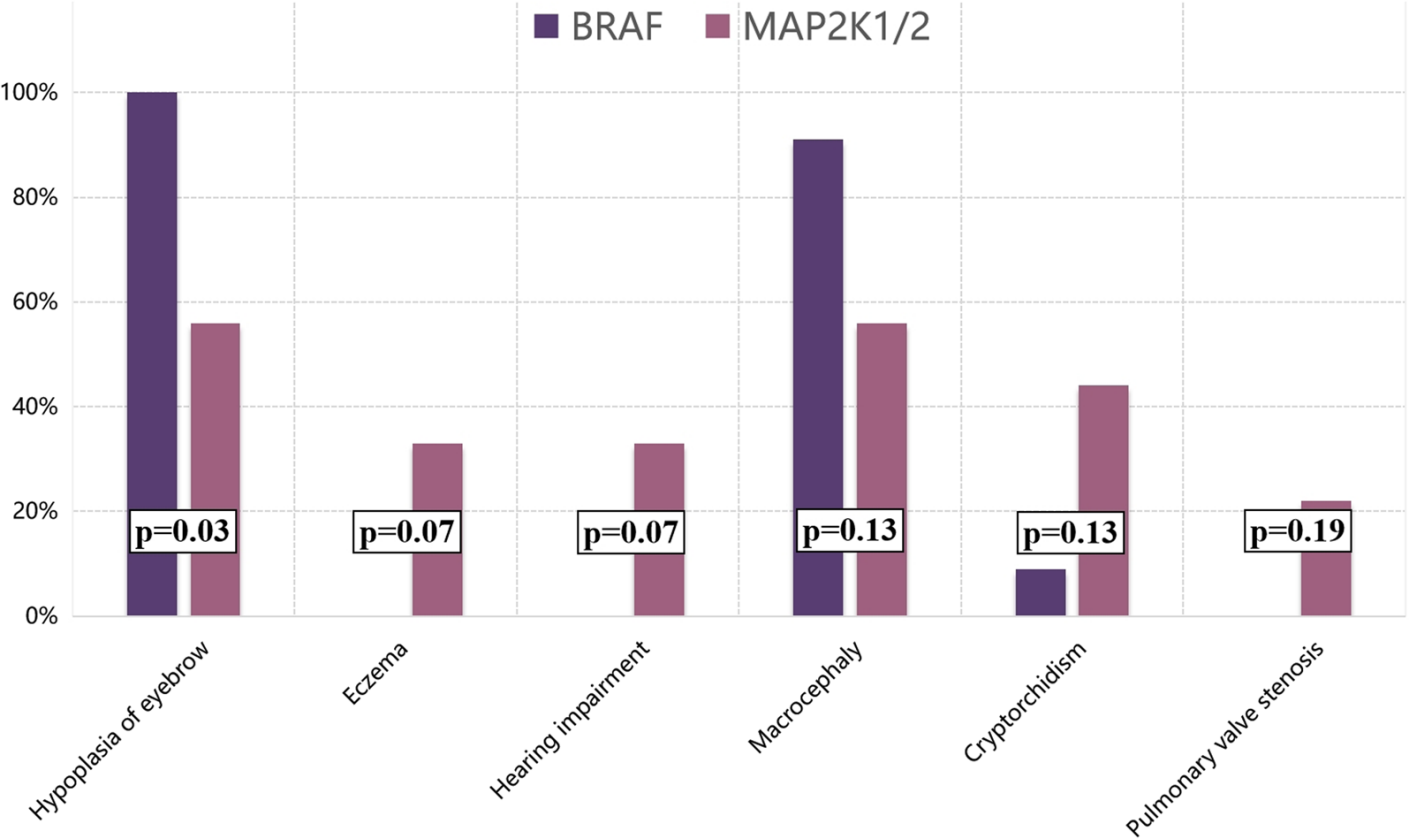
Genotype–phenotype correlation of CFC among BRAF and MAP2K1/2 mutation subgroup

Genotype–phenotype analysis was performed in CFC patients with variants in the *BRAF* or *MAP2K1/2* genes (Fig. 3). Individuals with *BRAF* variants exhibited a significantly higher rate of hypoplasia of eyebrows (100%) than those with variants in *MAP2K1/2* genes (56%; *p* = 0.03). Severe short stature occurred more frequently in patients with *BRAF* variants*,* although the difference was not statistically significant. Furthermore, eczema (*p* = 0.07), hearing impairment (*p* = 0.07), cryptorchidism (*p* = 0.13), and pulmonary valve stenosis (*p* = 0.19) were commonly found in patients with *MAP2K1/2* variants, while macrocephaly (*p* = 0.13) was often observed in patients with *BRAF* variants.

### Facial gestalt analysis of CFC

**Fig. 4:**
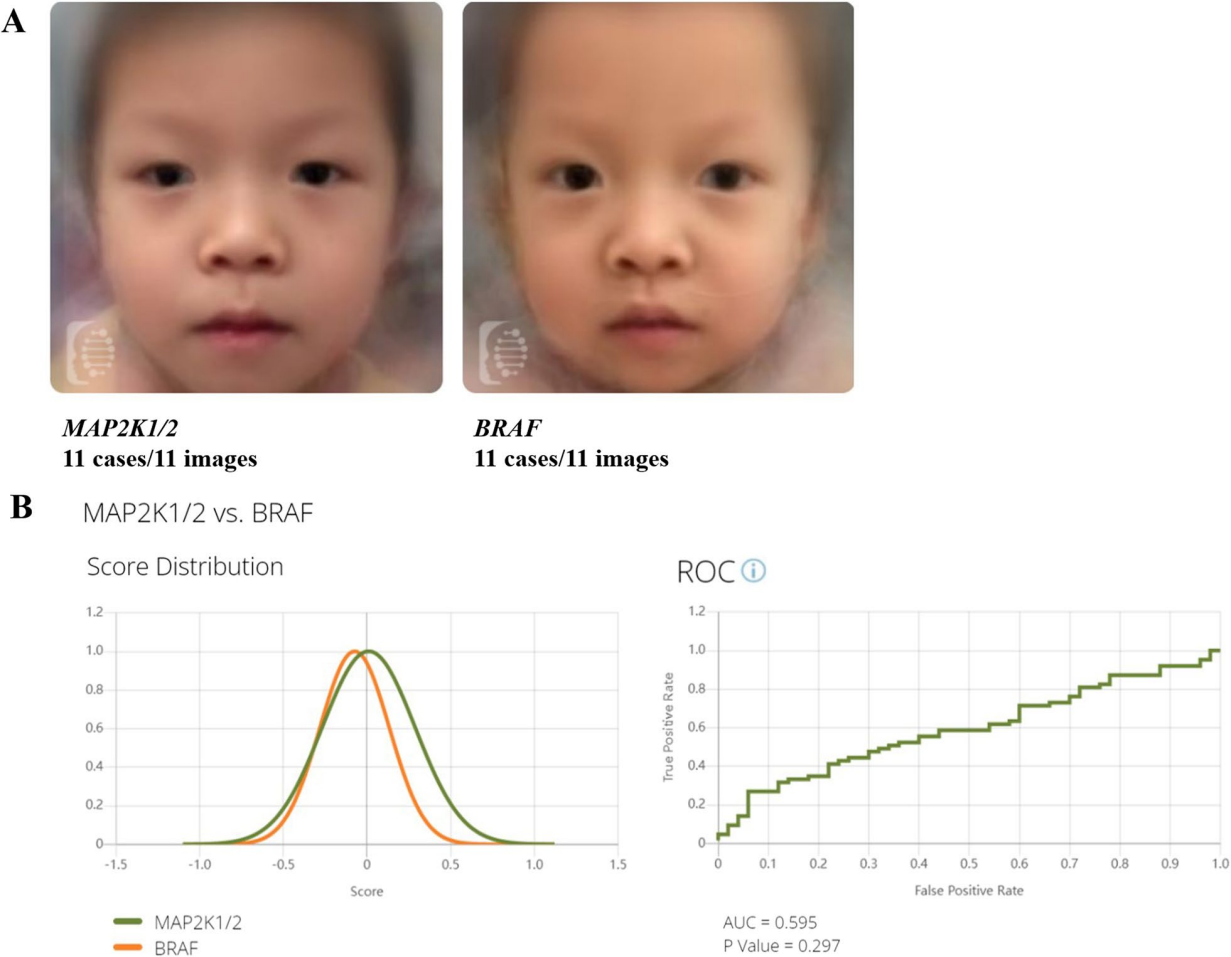
Facial analysis of patients. (**A**) Composite photos computed from images of BRAF and MAP2K1/2 mutation subgroup. (**B**) Score distribution and ROC curves of the comparison results of BRAF and MAP2K1/2 mutation subgroup

Pictures of the 20 Chinese CFC patients were collected and analyzed using Face2Gene CLINIC tools. For the majority of patients (18/20), CFC was listed in the TOP 5 diagnosis list produced by Face2Gene CLINIC tools. A composite photo was computed through Face2Gene RESEARCH (Fig. 4A). Furthermore, to assess patient facial patterns among different genotypes, we added two additional facial pictures of Chinese patients with *MAP2K1* variants [10, 11]. The receiver operating characteristic (ROC) curves showed that facial patterns were similar in CFC patients with different genotypes, with an area under the curve (AUC) of 0.595 (*p* = 0.297) (Fig. 4B). This suggests that facial recognition analysis cannot distinguish between different genotypes in patients with CFC.

## Discussion

In our study, NGS-based genetic was used to diagnose the majority of patients (19/20). NGS-based genetic testing is a rapid and inexpensive technique that provides a genetic, evidence-based diagnosis for CFC. Additionally, direct measurement of the head and face of CFC patients has been used to objectively enhance diagnostic accuracy [12]. In patients with moderate clinical features, artificial intelligence tools (Face2Gene) may help with facial recognition and improve diagnostic accuracy [13]; indeed, it has been reported that the mean accuracy computed by automated image analysis (Face2Gene RESEARCH) can help distinguish genotypes in NS patients [7, 13]. However, facial images could not differentiate between patients with variants in *BRAF* or *MAP2K1/2* genes in our study.

By comparing the phenotypes of 20 Chinese patients with CFC to those of a cohort of 186 non-Chinese CFC patients, we concluded that the frequencies of pulmonary valve stenosis, curly/sparse hair, epilepsy, and hypotonia were lower in Chinese patients (Table 2). The popularization of prenatal practice, ethnic differences, and the lifetime risk of epilepsy may explain these discrepancies. In contrast, the Chinese patients in our study more often experienced feeding difficulties, and patient 2 has had to receive prolonged supplemental feeding via nasogastric tube feeding since birth. This is likely because the majority of our patients (16/20) were diagnosed before the age of 3 (Additional file 1: Table S1). Feeding problems, including gastroesophageal reflux, suck/swallow dysfunction, and oral aversion, are commonly observed in CFC [1].

After analyzing the phenotypic diversity between patients with *BRAF* or *MAP2K1/2* variants, we found that the occurrence rates of hypoplasia of the eyebrows and macrocephaly were higher in patients with *BRAF* variants, whereas eczema, hearing impairment, cryptorchidism, and pulmonary valve stenosis were more common in patients with *MAP2K1/2* variants. Hypoplasia of the eyebrows was found to be of important significance in differentiating between *BRAF* and *MAP2K1/1* mutant patients (*p* = 0.03). In addition, severe short stature (height SD < − 3.0) was more common in individuals with *BRAF* variants than in those with *MAP2K1/2* variants. Retrospective studies have identified several other genotype–phenotype correlations. For example, the frequency and severity of epilepsies were higher in individuals with *BRAF* or *MAP2K1* variants (especially variants in the catalytic protein kinase domain of *BRAF* and p.Y130 site of *MAP2K1*) than in those with *MAP2K2* variants [14]. *BRAF* variants are also associated with distinct dermatological manifestations [15]. *MAP2K1* variants are correlated with skeletal deformities and motor dysfunction, and *MAP2K2* and *KRAS* variants are correlated with mild intellectual disability [1, 14].

Precise identification of subgroups among RASopathies has been an area of high interest and a bottleneck problem in previous studies. A recent cohort study found that CFC patients tend to have prominent problems in the nervous system, psychomotor development, and dermatologic manifestations, including severe intellectual disability, intractable epilepsy, spare/curly hair, hypoplasia of the eyebrow, and melanocytic nevi [16]. Neurological, dermatologic, and gastrointestinal issues and severe short stature tend to be less common in patients with NS than in those with CS or CFC [17]. Outstanding perinatal problems, such as hypoglycemia, arrhythmia, coarse face, intractable feeding difficulty, and a higher risk of tumors, are more common in patients with CS [17].

*BRAF* is the most common pathogenic gene of CFC. Variants in *BRAF* are distributed in exons 6 and 11–17; the most frequent missense variant is Q257R in exon 6 (29%), followed by E501G (12%) and G469E (6%) in exons 12 and 11, respectively [3]. The variant spectrum of *BRAF* in our study (Fig. 2) has been reported previously and included the hot spot variants Q257R (6/20) and E501G (1/20), as well as relatively rare variants D638E (2/20), Q262K (1/20), and T244P (1/20). Variants in *MAP2K1* occur in exons 2, 3, and 6 (Y130C is the most frequent variant), while those in *MAP2K2* occur in exons 2, 3, and 7 [11, 18]. We found two novel missense variants in *MAP2K1/2* in our study, L54P (*MAP2K1*) and D140H (*MAP2K2*), both of which are located in the functional domain of the protein and were predicted to be deleterious using three bioinformatics tools (Table 3).

Short stature is a common feature in all RASopathies, and two-thirds of patients with CFC have short stature. This is likely because the RAS/MAPK pathway contributes to intracellular insulin-like growth factor (IGF-1) signaling, and MAPK activation is important for the synthesis and release of GH [1]. Feeding difficulties and gastrointestinal abnormalities can also cause short stature [19]. In a recent study, seven patients with CFC underwent GH treatment and height SDS increased by more than 1 SD after two years of GH therapy without side effects [16]. Four of the 20 Chinese patients in our study were treated with GH; patient 4 (GH deficiency) and patient 6 achieved a satisfactory result following GH treatment, with a 10–11 cm increase in height during first 6 months. Patient 18 underwent three months of GH therapy with no change in height. GH therapy data for patient 13 were not available. None of the patients experienced side effects from GH treatment, demonstrating that CFC should be screened for GH deficiency firstly and then be assessed the possibility of GH treatment.

RAS is the most frequently mutated oncogene in human cancers, with variants occurring in approximately 30% of all cancers [20]. Somatic mutations of *BRAF* have been associated with various malignancies including malignant melanoma, thyroid, lung, ovarian and colorectal cancers [21]. Conversely, germline variants in the *BRAF* gene rarely cause cancer, except in a few cases of acute lymphoblastic leukemia and non-Hodgkin’s lymphoma. For patients carrying both germline and somatic variants in the *BRAF* gene, regular monitoring of the tumor is necessary [16]. In our cohort, patient 12 was diagnosed with Langerhans cell histocytosis when he was 2.4 years old. He was cured after chemotherapy in our hospital, and his tumor indicators were normal during long-term follow-up. Among RASopathies, CS patients were reported to carry the highest risk of malignancy (10.8%), followed by NS patients (3.9%), and CFC patients (3.5%) [21, 22]. Even so, tumor risk should be monitored, especially when GH is administered.

Our study was limited by the lack of CFC patients with *KRAS* variants in our cohort and the low number of patients with *MAP2K2* variants (2/20), which constrained our assessment of genotype–phenotype correlations. Additionally, the categories of phenotypes selected in our study were not completely consistent with those of the 186 non-Chinese patients; therefore, we only analyzed the phenotypes observed in both cohorts to obtain detailed phenotypic diversity between different ethnic CFC patients.

## Conclusions

Herein, we reported the molecular and phenotypic spectra of 20 Chinese patients with CFC, including the discovery of two novel variants. The CFC phenotype in Chinese populations overlaps with that in non-Chinese populations, although some phenotypic differences exist. Diversity of phenotype was observed between patients with *BRAF* and *MAP2K1/2* variants, and automated facial image analysis tools (Face2Gene) failed to distinguish CFC patients by genotype. Overall, we conclude that the diagnosis of CFC can be facilitated using typical clinical features and facial recognition software tools and recommend genetic testing using NGS-based approaches for final diagnosis.

## Methods

### Patients

This study was approved by the ethics committee of the Shanghai Children's Medical Center, Shanghai Jiaotong University School of Medicine (Shanghai, China). Patients were recruited from the Shanghai Children's Medical Center and other domestic third-class hospitals between 2016 and 2022. CFC patients with a genetic diagnosis of *MAP2K2* variant were directly included in the study, whereas those who exhibited other RASopathy manifestations with a genetic diagnosis of *BRAF, MAP2K1,* or *KRAS* variants were excluded. Facial pictures, medical history, and clinical features were collected through electronic surveys and confirmed by telephone interviews and face-to-face diagnosis. All physicians were pediatricians with additional subspecialty training in endocrinology. Careful counseling was provided to all patients and their parents to obtain informed consent.

We used the human phenotype ontology (HPO, https://hpo.jax.org/app/) to label the phenotype of CFC patients with a unique HPO number. The phenotypes we collected from each patient were compared to those of 186 non-Chinese patients with CFC [5].

### Molecular genetic analysis

All patients underwent whole exome sequencing, except for patient 6, who was tested for a panel of hereditary liver diseases. Two milliliters of peripheral blood were collected from patients and their parents. Blood samples were stored in ethylenediaminetetraacetic acid anticoagulant tubes for examination. Exons were captured using the Agilent Sure Select method. High-throughput sequencing was performed using the Illumina sequencing platform, sequencing data were matched and analyzed using NextGENe® software, and variation filtering and interpretation were performed using the Ingenuity online software system. All detected variants were confirmed by Sanger sequencing in both parents. The method recommended by the ACMG was used to classify the detected variants. To determine the potential pathogenicity of the novel missense variants, three in silico prediction methods were used: Mutation Taster (http://www.mutationtaster.org), Sorting Tolerant from Intolerant (SIFT; http://sift.jcvi.org/), and Polymorphism Phenotyping v2 (PolyPhen-2; http://genetics.bwh.harvard.edu/pph2/).

### Automated image analysis

An AI approach was used to analyze facial images of patients with CFC. We use Face2Gene (F2G Inc., Boston; http://www.fdna.com/face2gene/) to evaluate the identification accuracy of CFC patients, which analyzes phenotypic traits based on pattern recognition of frontal photographs of the patients. Face2Gene CLINIC tools were used to analyze the diagnosis accuracy of CFC from only facial pictures, and the diversity of facial patterns between different genotypes was computed using RESEARCH tools.

### Statistics analysis

The prevalence of each phenotype was analyzed using the chi-squared (χ^2^) test. Fisher's exact tests were performed to compare the incidences of the various phenotypes between Chinese and non-Chinese patients. Phenotypic differences between the genotypes were also analyzed by chi-squared (χ^2^) test. *p* values < 0.05 were considered statistically significant. All analyses were conducted using the statistical software package IBM SPSS Statistics version 26.

### Supplementary Information

**Additional file 1: Table S1.** Clinical phenotypes of the CFC patients with germline BRAF, MAP2K1, MAP2K2 mutations.

## Data Availability

Not applicable.
