# Supplementary material for: Molecular and phenotypic spectrum of cardio-facio-cutaneous syndrome in Chinese patients
Source: Orphanet J Rare Dis. 2023 Sep 11;18:284. doi: 10.1186/s13023-023-02878-0 (PMC10496309; doi:10.1186/s13023-023-02878-0)
Supplement: Supplementary file 1 — Additional file 1: Table S1. Clinical phenotypes of the CFC patients with germline BRAF, MAP2K1, MAP2K2 mutations. [file 13023_2023_2878_MOESM1_ESM.docx]

| Patient  no. | Mutation | Sex | Age(yr) | | Height SDS | GH  therapy | CHD |  |  |  |  |  |  |  |  |  |  |  |  |  |  |  |  |
| --- | --- | --- | --- | --- | --- | --- | --- | --- | --- | --- | --- | --- | --- | --- | --- | --- | --- | --- | --- | --- | --- | --- | --- |
|  |  |  | At diagnosis | Current |  |  |  | Macro-  cephaly | Curly/sparse hair | Eyebrow | Ocular | Oro-nasal findings | Ears | Dermatologic findings | Abnormal nail growth | ID | Hypotonia | Feeding difficulties | Skeletal anomalies | Cryptorchidism | Neoplasm | [Perinatal](javascript:;) findings | Other findings |
| 1 | BRAF | M | 7m | 4y6m | -8.3 | - | HCM | + | -/+ | HY | EP | WNB, AN | LSE | MN, KP | + | + | - | + | - | - | - | Ph, INT, PB | Cafe-au-lait spot, SD, BMA |
| 2 | BRAF | F | 5m | 7m | -4.1 | - | ASD | - | -/+ | HY | - | WNB, AN | - | MN, KP, HE | - | NA | - | + | PE | - | - |  | BMA |
| 3 | BRAF | F | 2y2m | 6y2m | -1.5 | - | - | + | +/+ | HY | DPF, ST | AN, AUM | LSE | MN | + | + | + | + | PE, PP | - | - | Ph | SD, BMA |
| 4 | BRAF | M | 1y1m | 6y9m | -6.2 | + | ASD | + | -/+ | UO, HY | DPF, EP | WNB | LSE | MN | + | + | - | + | SC, PE, PP | - | - | AFM, FP | SD |
| 5 | BRAF | M | 6m | 2y1m | -0.4 | - | PFO | + | -/+ | UO, HY | - | WNB, DM | LSE | MN, PH | + | NA | - | + | PE | - | - | Ph |  |
| 6 | BRAF | F | 9m | 4y3m | -2.8 | + | - | + | +/+ | HY | DPF, EP | WNB, AN | LSE | MN | - | + | + | + | PE | - | - | Ph, PB | BMA |
| 7 | BRAF | F | 9m | 5y2m | -2.5 | - | - | + | +/+ | HY | ST | DM | - | MN, HE | + | + | - | + | SC, JH | - | - | Ph, PB | SD |
| 8 | BRAF | F | 7m | 2y | -3.4 | - | HCM | + | -/- | HY | - | AN | LSE | MN | - | NA | + | + | - | - | - | Ph, AFM, PB | SD |
| 9 | BRAF | F | 1y | 2y7m | -1.9 | - | - | + | +/+ | HY | EP | AN | - | - | - | NA | + | + | - | - | - |  | SD |
| 10 | BRAF | M | 2y9m | 4y6m | -4.3 | - | ASD | + | -/+ | HY | EP, ST | WNB, AN, DM | LSE | HE | - | + | + | + | PC | - | - | Ph | SD, BMA |
| 11 | BRAF | M | 4y2m | 5y7m | -4.3 | - | PFO | + | +/- | HY | PT, DPF, EP, ST | WNB, AN, WM | LSE | - | - | + | + | + | PC | + | - | Ph, AFM, FP, PB | SD |
| 12 | MAP2K1 | M | 5y3m | 9y6m | -2.7 | - | - | - | -/- | - | DPF, EP | WM, DM | LSE | MN, EC | - | + | - | + | SC | + | + (LCH) |  |  |
| 13 | MAP2K1 | M | 1y4m | 3y11m | -4.3 | + | ASD | + | -/+ | UO, HY | EP | AN | - | KP, HE | - | + | + | + | SC, PP | + | - |  | SD, BMA |
| 14 | MAP2K1 | M | 7m | 5y10m | -4.8 | - | PVS ASD | - | -/- | - | DPF, EP | WM, DM | - | MN, HE | - | + | - | + | PE, PP | - | - | INT | SD, BMA |
| 15 | MAP2K1 | F | 5m | 2y4m | -0.3 | - | ASD | + | -/+ | HY | PT, EP, ST | WNB, AN | LSE, HI | KP, HE | + | NA | - | + | PE, PP | - | - | Ph, INT, AFM, PB | SD, BMA |
| 16 | MAP2K1 | F | 2y2m | 2y5m | -1.4 | - | PFO | - | -/- | - | - | WNB | LSE | - | - | NA | - | + | - | - | - | PB |  |
| 17 | MAP2K1 | M | 8m | 11m | -2.5 | - | - | + | +/+ | HY | DPF, ST | AN | - | - | + | NA | + | + | PC | - | - | Ph | SD |
| 18 | MAP2K1 | M | 5y2m | 8y2m | -4.7 | + | PFO | + | -/- | UO, HY | EP, DPF, PT | AN | LSE | MN, KP, EC | + | + | - | - | PE, PP | + | - | Ph | BMA |
| 19 | MAP2K2 | M | 4y9m | 5y2m | -0.3 | - | PVS  ASD | + | +/+ | UO, HY | ST | AN | LSE, HI | MN, KP, HE, EC | + | + | + | + | PC, PP, JH | + | - | Ph | BMA |
| 20 | MAP2K2 | F | 1y2m | 1y3m | +0.5 | - | - | - | -/+ | - | ST | AN | HI | - | + | NA | + | + | - | - | - | PB | epilepsy, SD, BMA |

Supplementary Table 1 Clinical phenotypes of the CFC patients with germline BRAF, MAP2K1, MAP2K2 mutations

no. number, yr year, NA not applicable, SDS standard-deviation score, GH growth hormone, CHD congenital heart disease, HCM hypertrophic cardiomyopathy, ASD atrial septal defect, PFO patent foramen ovale, PVS pulmonary valve stenosis, UO ulerythema ophryogenesis, HY hypoplasia, DPF downslanted palpebral fissures, ST [strabismus](javascript:;), EP epicanthus, PT ptosis, WNB wide nasal base, AN anteverted nares, WM wide mouth, AUM abnormal uvula morphology, LSE low-set ears, HI hearing impairment, MN melanocytic nevi, KP keratosis pilaris, HE hemangioma, PH palmoplantar hyperkeratosis, SC scoliosis, PC pectus carinatum, PE pectus excavatum, PP pes planus, JH joint hypermobility, Ph Polyhydramnios, INT increased nuchal translucency, AFM abnormal fetal morphology, FP fetal pyelectasis, PB premature birth, DM dental malocclusion, EC eczema, SD sleep disturbance, Sx symptoms, BMA brain MRI abnormality
